# Case of Facial and Palatine Paralysis and Loss of Equilibrium, Produced by a Fall on the Head

**Published:** 1881-02

**Authors:** M. Putnam Jacobi

**Affiliations:** 101 W. 34th street, New York


ARTICLE IV.

### CASE OF FACIAL AND PALATINE PARALYSIS, AND LOSS OF EQUILIBRIUM, PRODUCED BY A FALL ON THE HEAD.

BY M. PUTNAM JACOBI, M. D., NEW YORK.

Ralph Rosenstein, aged 2 years, was brought to the dispensary of the Mt. Sinai Hospital, on October 18, 1880; the mother stated that a week previous he had fallen off a chair to the floor, striking the back of his head. No especial effect from the fall was, however, observed during the two days first following the accident, but on the third day began to droop; he allowed his head to fall forward, walked with a staggering gait, and finally refused altogether to walk, or even to stand. Previous to the fall, he was said to have walked well. Co-incident with the symptoms, he began to cough.

On the day of examination it was found that he could stand; could move his legs while supported in a standing position, and very freely while in bed; but would not stir from his place, even to follow the mother who pretended to lead the way. He then burst out crying, and sat down on the floor, but seemed unable to try to walk. During the examination he asked for water, and as he was drinking it was noticed that the water regurgitated through the nose. On inquiry it appeared that this had happened ever since his fall, but the mother had not thought it worth mentioning. No diphtheria existed or had existed to explain this paralysis of the soft palate. The uvula was markedly deviated towards the left. The right angle of the mouth drooped; thus there was evidently paralysis of lower branches of the right facial nerve, the staphylo palatine and buccal branches. But the upper branches were intact: the eyelids and the muscle of the forehead presented a perfectly normal appearance. There was no deviation of the tongue; none of the eyeball; the pupils were unaffected. From the tender age of the child it was impossible to ascertain whether any deafness exisited on the right side or whether there was any sensation of vertigo. The morbid symptoms consisted therefore in paralysis of the right facial nerve and in loss of power to maintain equilibrium in an upright position without paralysis of the lower extremities. The difficulty of walking seemed to be entirely due to dread of falling.

Two localities suggested themselves as the seat of a lesion capable of explaining this group of symptoms. The first, some portion of the petrous bone that might include at once the facial nerve and one of the semi-circular canals of the internal ear.

It is well known at present that lesions of these canals seriously interfere with maintenance of equilibrium of the body. Experiments have even determined the direction in which each canal seems to exercise an influence. Crum Brown* has so analyzed these influences as to bring them within the same general law as that of the co-ordinating centres in the cerebellum. According to this law, the inclination of the body in any given direction tends to excite the centre situated on the opposite side of the body in such a manner that the complete falling over is prevented by antagonism. In the labyrinthimic canals, according to a plausible hypothesis, the nerves are stimulated when the endolymph flows in excess into the ampulla at one extremity, the motion of the fluid being freey determined by the position of the head. The horizontal canals are all situated in the same plane, but their respective ampullæ are turned in different directions. When the head inclines to the right side, the endolymph flows from the ampullæ into the canal; while at the same time on the left side it is flowing from the canal into the ampulla. The ampullary expansion of the left auditory nerve is therefore stimulated by the excess of pressure, and an impression conveyed to co-ordinating centres in the cerebellum, which tends to restore equilibrium. Destruction of the horizontal canal on the left side should therefore be followed by a tendency to fall to the right, from loss of the antagonistic mechanism. According to Goltz (quoted by Ferrier, "Functions of the brain,") division of the auditory nerve will, in the frog, be followed by the same symptoms as division of the semi-circular canals. This latter fact alone could explain the circumstances of our case, supposing that the loss of equilibrium were due to injury of the petrous nerve. For although the acqueductus Fallopii carrying the facial nerve passes almost between the cochlea and the semi-circular canals above the vestibule of the inner ear, yet a lesion common to those can`ls and to the facial nerve, would necessitate a fracture of the bony floor of that acqueduct. The slight nature of the injury sustained by the child, and the transciency of its effects both precluded the supposition of so grave a lesion.

Again: as the lesion of the facial was certainly on the right side, injury to to the semi-circular canals, if existing at all, must have been on the right side also. But, as already stated, injury of the horizontal canal on the right side, is followed by a tendency to fall on the left, on account of loss of the mechanism which naturally compensates

Journal of Anatomy and Physiology, May, 1874.

a tendency to fall on the left. In our case the child always fell on the right.

This fact might at first seem to annul altogether the hypothesis of the petrous bone lesion. The fact that the upper facial remained intact might seem compatible with any form of peripheric lesion, and necessitate reference to the centres. The portion of the encephalon whose injury would be capable of inducing paralysis of the right facial nerve, and loss of equilibrium, is the inferior surface of the right lateral lobe of the cerebellum, near the median lobe, and also near the facial nerve after its emergence from the medulla.

"The maintenance of equilibrium is an example of adaptive, responsive or asthetiko-kinetic action, depending on the co-ordination in some central organ of certain afferent impressions with special motor adjustments. The afferent factors of this mechanism are mainly of three kinds, namely, tactile, visual, and labyrinthic impressions. We are justified in concluding that the cerebellum is the central organ of this co-ordination." (Ferrier, p. 113).

According to Nothnagel (Topische Hiru kraukheiten,) in co-ordination of movements and loss of equilibrium are the only characteristic signs of lesion of the cerebellum.* This is shown with special vacillation of gait, and with severe vertigo. Nothnagel adds, that if this symptom exist in cerebellar disease, it indicates a direct or indirect affection of the middle lobe of the cerebellum. According to the same authority, paresis or paralysis of the facial nerve is occasionally, although rarely observed, in lesions of the cerebellum: and is then always due to pressure upon the nerve after its emergence from the medulla, by means of some lesion, as a hemorrhage or tumor, sufficiently near the surface of the cerebellum to exercise an extra cerebellar effect. In such cases the paralysis resembles that due to lesions of the motor tract of the cerebrum in being confined to the buccal branches of the facial.

Such a paralysis would therefore not correspond to that observed in our case, where the buccal lesion was much less prominent than that of the palatine branches of the portio dura. These branches are derived from the greater petrous nerve which is given off from the facial at the geniculate ganglion." (Longet.) The geniculate ganglion is situated at the first angle of the acqueductus Fallopii, and

Of course, as the author especially notices, this symptom is not exclusive to the cerebellum.

thus lesions of the portio dura, during their passage through this acqueduct are especially liable to be attended by paralysis of the palate; a comparatively rare sequence of either completely central or completely peripheric lesion of the nerve.

The distance is very small from the geniculate ganglion to the internal meatus, where the auditory nerve still lies side by side with the facial. It is conceivable that a slight hemorrhage should occupy all this space, and thus co-incidently affect the auditory nerve, the facial, and the branches emanating from its geniculate ganglion.

The experiments of Goltz, already quoted, which, showing that section of the auditory nerve may have the same effect as lesion of the semi-circular canals, would explain why a lesion in the vicinity thus defined should occasion loss of equilibrium. The auditory nerve, though it represents neither the organ receiving labyrinthic impressions nor the central organ receiving them, unquestionably constitutes the path by which they are conveyed to that central organ, the cerebellum. A portion of the roots of the auditory nerve pass to the cerebellum in the restiform bodies, (Meyner) so that each auditory nerve is connected with the lateral hemisphere on the same side.

According to Ferrier, experimental lesions of the lateral lobes of the cerebellum, whether destructive or irritative, are followed by the same results as are lesions of the peduncles. The displacement of the body is sometimes toward the side of the lesions, sometimes toward the opposite side. The latter, observes Ferrier, is more likely to occur when the lesion is limited, the former when it is extensive.

Hitzig, (untersuch uber das Gehirn, p. 203) pointed out that the passage of a galvanic current through the cranium was followed by a sudden sinking of the head towards the side on which the irritation is applied, i. e., where the anode is placed. Now an irritation transmitted to the lateral lobe of the cerebellum, along the auditory nerve, should have the same effect as this electrical irritation. Equilibrium would be disturbed from the unequal stimulus of the co-ordinating centres, and, as experiment shows, without clearly explaining why, the tendency might be to fall on the same side as the lesion. The case differs from that of lesion of the labyrinthic canals, affecting the terminal expansion of the nerve, because that implies destruction of a mechanism by which a tendency to fall towards the other side is habitually compensated, whereas, lesion of the trunk of the nerve coincides in effect with lesion of the lateral lobe of the cerebellum on the same side.

From the foregoing reasons we have ventured to diagnose a hemorrhage, extending from the internal meatus through the acqueductus Fallopii as far as the geniculate ganglion, as the cause of the symptoms observed in the case of Ralph Rosenstein.

M. Putnam Jacobi, M. D,

101 W. 34th street, New York.

